# The ethical experiences of trainees on short-term international trips: a systematic qualitative synthesis

**DOI:** 10.1186/s12909-018-1424-7

**Published:** 2018-12-29

**Authors:** James Aluri, Dane Moran, Antony G. Kironji, Bryn Carroll, Jacob Cox, Chi Chiung Grace Chen, Matthew DeCamp

**Affiliations:** 1 Department of Psychiatry, Johns Hopkins Hospital, Meyer 4-181, 600 N. Wolfe Street, Baltimore, MD 21287-7381 USA; 2 Department of Emergency Medicine, Baylor College of Medicine, Houston, Texas USA; 3 Department of Emergency Medicine, Boston University Medical Campus, Boston, Massachusetts USA; 4 Department of Pediatrics, Children’s Hospital of Philadelphia, Philadelphia, PA USA; 5 Department of Medicine, Newton-Wellesley Hospital, Boston, MA USA; 6 Department of Ophthalmology, Massachusetts Eye and Ear Infirmary, Boston, MA USA; 7 Department of Gynecology and Obstetrics, Johns Hopkins Hospital, Baltimore, MD USA; 8 Berman Institute of Bioethics and Division of General Internal Medicine, Johns Hopkins University School of Medicine, Baltimore, MD USA

**Keywords:** Medical education, Global health, Medical ethics, International health, Medical missions

## Abstract

**Background:**

Medical student and resident participation in short-term international trips for trainees (STINTTs) has increased in the past few decades. However, there has been no systematic review of trainees’ actual ethical experiences. The authors sought to identify what ethical issues medical trainees encounter during STINTTs, as elicited by and reported in peer-reviewed, quantitative and qualitative research papers.

**Methods:**

The authors systematically searched five academic databases finding 659 unique titles and abstracts. The authors applied inclusion and exclusion criteria to these titles and abstracts resulting in fourteen papers, which were analyzed using qualitative thematic synthesis.

**Results:**

The qualitative analysis of the papers generated four themes: (1) Trainees’ Concerns Over Perpetuating Medical Tourism; (2) Struggling to Identify and Balance the Benefits and Harms of STINTTs; (3) The Complicated Trainee Mens (mind); and (4) Ethical Situations Encountered by Trainees. The fourth theme, which was the largest, was further divided into (a) Navigating social and cultural dynamics, (b) Trainees’ experiences related to the learner role, and (c) Ethical situations not qualifying for other catagories. Some of these issues reported in the empirical research papers are well represented in the broader literature on STINTTs, while others were less so—such as mistreatment of trainees. All included papers were published after 2010, and comprised a total of less than 170 medical trainees.

**Conclusions:**

Medical trainees report experiencing a wide range of ethical challenges during short-term international trips in which they engage in clinical or research activities. The authors call educators’ attention to specific challenges that trainees face. The relevant literature covering US and Canadian STINTTs is relatively young and largely qualitative. The authors briefly sketch a program for expanding the research on this increasingly common educational experience.

## Background

Medical trainees increasingly participate in short-term international trips. U.S. medical student participation in global health experiences has increased from 6% in 1984 to around 30% in 2010 [1]. In the absence of an established definition, we consider medical short-term international trips to be any clinical or research experience lasting less than 3 months that took place in another country (often low- or middle-income settings). Recognizing that no accepted definition of “short-term” exists, the choice of 3 months attempts to strike a balance among competing definitions. As examples, two prior reviews on short-term international trips (not specific to trainees) define the maximum length of short term trips as 4 and 8 weeks [2, 3], a recent position paper allows for up to several months, [4] and seasoned global health professionals might consider anything less than 6 or even 12 months as short-term [5, 6].

We further characterize these trips by the types of participants and organizational affiliation. For example, some trips are organized by non-governmental agencies for fully trained physicians. In this paper, we focus on short-term international trips for trainees (STINTTs)—short-term international trips developed by a medical school or residency program for the purpose of trainee education. Other purposes include, but are not limited to, delivery of health care services, building local health care capacity, the development of collaborative partnerships, and research, among others. We stake no claims here as to which among these purposes is most important.

In the past several years, significant attention has been given to the ethical dimension of STINTTs and to developing best practice guidelines when ethical challenges arise [5]. Some papers in the existing literature note several positive aspects of STINTTs—including the development of clinical, cultural, and language skills; increased understanding of healthcare systems and tropical diseases; and personal and moral growth [7, 8].

Conversely, the literature also raises several ethical concerns—including the attitudes of trainees, trainees’ impact on host resources, the challenges of negotiating cultural differences, the safety of trainees and patients, short-sighted interventions, poor supervision of trainees, and possible neo-colonialist frameworks that value the priorities of the Western institutions over hosting institutions in developing nations [1, 5, 8–17]. Some of the ethics literature describes case studies or individual experiences of ethical challenges abroad [17–20]. Others have re-framed the understanding of STINTTs to encourage trainees to approach the trips in terms of virtues [21] or have suggested expanding the ethical discourse beyond discussion of outcomes related to goods and services [11].

Primary research studies [1, 22, 23] have slowly started to fill the identified gap of literature documenting the ethical experiences of medical trainees on STINTTs [5, 24]. Nonetheless, systematic reviews of the literature on short-term international trips have noted the lack of a robust research base. One review found that the majority (78%) of the literature on short-term medical ‘missions’ was descriptive, [2] and another pointed out the dearth of high-quality empirical studies on medical short-term international trips [3]. Neither was specific for STINTTs, whose participants (because of their trainee status) might face unique ethical challenges.

To our knowledge, only a single systematic review exists on the ethics of STINTTs. This review characterized the educational interventions that have been developed to prepare medical students for the ethical challenges on these experiences and described thirteen ethical situations that students might encounter on STINTTs [25]. Yet none of the published papers included in their final analysis were research studies of trainees’ actual experiences.

Recognizing this reported gap in the literature and the need for educators to match ethics preparation with what trainees are experiencing abroad, we formulated the research question: “what does the peer-reviewed, quantitative and qualitative literature document regarding medical trainees’ ethical experiences on STINTTs?”

## Methods

To answer our research question, we conducted a systematic qualitative synthesis—a type of systematic review that synthesizes qualitative results in the existing literature.

The reporting of our methods borrowed elements (such as a flow chart of the search results) from both the PRISMA guidelines for systematic reviews [26, 27] as well as the ENTREQ (Enhancing transparency in reporting the synthesis of qualitative research) [28]. This blended approach sought to glean the structure of PRISMA reporting for our literature search, while deferring to the ENTREQ’s suitability for qualitative synthesis of the resulting papers. This hybrid approach also reflected the natural evolution of the project which we initiated under the quantitative-oriented paradigm of systematic reviews, and transitioned to a qualitative orientation after our search resulted in mostly mixed-methods and qualitative papers. For the ENTREQ checklist, see the Appendix.

### Eligibility criteria

To meet inclusion criteria, the paper had to address the ethical experiences of medical trainees involved with short term international trips. A paper’s relevance to “ethical experiences” was determined by the presence of the words, “ethics,” “bioethics,” “moral,” or related words in the paper. “Medical trainees” were defined as students at a medical training programs (both undergraduate and graduate) in the U.S. or Canada accredited by the Liaison Committee on Medical Education (LCME), Committee on Accreditation of Canadian Medical Schools (CACMS), Accreditation Council for Graduate Medical Education (ACGME), or the Royal College of Physicians and Surgeons of Canada (RCPSC). “Short term international trips” had to take place outside the U.S. or Canada and included any clinical or research-based trip for less than 3 months.

We restricted our search to papers that used research methodology (e.g. surveys, qualitative methods, or mixed-methods research) to elicit these experiences directly from trainees. Therefore, anecdotal stories and case reports would be excluded, unless the paper clearly indicated how the experiences or cases were elicited from trainees. Additional exclusion criteria included being published in a language other than English and lack of full text availability (e.g. lists of conference abstracts). Papers that interviewed faculty members, host supervisors, mentors, or others were included so long as they addressed the lived ethical experiences that medical learners faced.

### Information sources and search

With a medical informationist, we translated our targeted content into search phrases for five databases: Pubmed, Embase, Education Source, Academic Search Complete, and Web of Science (Core Collection). For our full search strategy see Table 1. To correct for narrow search terms and new publications, we also allowed for the inclusion of papers that met our inclusion criteria, but were not revealed through the search strategy. Such papers were collected through reference searches, journal notices, or personal reading.

**Table 1:** Search Terms

Database	Query
Pubmed	(“ethics”[Subheading] OR “ethics”[All Fields] OR “ethics”[MeSH Terms] OR Bioethics [tiab] OR bioethical[tiab] OR ethical [tiab] OR ethically [tiab] OR moral [tiab] OR morals [tiab] OR morally [tiab] OR morality[tiab] or unethical[tiab] or immoral[tiab]) AND (“Global Health”[Mesh] OR “global health”[tiab] or “international health”[tiab] OR “global setting”[tiab]) AND (“Education, Medical”[Mesh] OR “medical education” OR “medical students” OR “curriculum” OR “predeparture training” OR “training”[tiab] OR “trainees”[tiab] OR “undergraduate”[tiab] OR “Curricula”[tiab] OR “Academic program”[tiab] OR “Academic programs”[tiab] OR “Course”[tiab] OR “Pedagogy”[tiab] OR “Clinical education”[tiab] OR “Workshop”[tiab] OR “Educational”[tiab])
Embase	(‘ethics’/exp. OR ‘bioethics’/exp. OR ‘medical ethics’/exp. OR “ethics” OR Bioethics:ab, ti OR bioethical:ab, ti OR ethical:ab, ti OR ethically:ab, ti OR moral:ab, ti OR morals:ab, ti OR morally:ab, ti OR morality:ab, ti or unethical:ab, ti or immoral:ab, ti) AND (“global health”:ab, ti or “international health”:ab, ti OR “global setting”:ab, ti) AND (‘medical education’/exp. OR “medical education” OR “medical students” OR “curriculum” OR “predeparture training” OR “training”:ab,ti OR “trainees”:ab, ti OR “undergraduate”:ab,ti OR “Curricula”:ab, ti OR “Academic program”:ab, ti OR “Academic programs”:ab, ti OR “Course”:ab, ti OR “Pedagogy”:ab, ti OR “Clinical education”:ab, ti OR “Workshop”:ab, ti OR “Educational”:ab, ti)
Education Source	(DE “Ethics -- Study & teaching” OR DE “Bioethics -- Study & teaching” OR “ethics” OR Bioethics OR bioethical OR ethical OR ethically OR moral OR morals OR morally OR morality or unethical or immoral) AND (“global health” or “international health” OR “global setting”) AND (DE “MEDICAL education” OR DE “MEDICAL students” OR “medical education” OR “medical students” OR “curriculum” OR “predeparture training” OR “training” OR “trainees” OR “undergraduate” OR “Curricula” OR “Academic program” OR “Academic programs” OR “Course” OR “Pedagogy” OR “Clinical education” OR “Workshop” OR “Educational”)
Academic Search Complete	(DE “BIOETHICS” OR DE “ETHICS” OR “ethics” OR Bioethics OR bioethical OR ethical OR ethically OR moral OR morals OR morally OR morality or unethical or immoral) AND (“global health” or “international health” OR “global setting”) AND (DE “MEDICAL education” OR DE “MEDICAL students” OR “medical education” OR “medical students” OR “curriculum” OR “predeparture training” OR “training” OR “trainees” OR “undergraduate” OR “Curricula” OR “Academic program” OR “Academic programs” OR “Course” OR “Pedagogy” OR “Clinical education” OR “Workshop” OR “Educational”)
Web of Science (Core Collection)	(“ethics” OR Bioethics OR bioethical OR ethical OR ethically OR moral OR morals OR morally OR morality or unethical or immoral) AND (“global health” or “international health” OR “global setting”) AND (“medical education” OR “medical students” OR “curriculum” OR “predeparture training” OR “training” OR “trainees” OR “undergraduate” OR “Curricula” OR “Academic program” OR “Academic programs” OR “Course” OR “Pedagogy” OR “Clinical education” OR “Workshop” OR “Educational”)

The full search terms, as entered, are included above for each of the five databases

### Study selection

The search resulted in 659 unique titles. Two independent screeners reviewed titles and abstracts of all search results. Disagreements were adjudicated by discussion between the two screeners. When still unresolved (this occurred for 58 of the 659 titles and abstracts), the principal investigator arbitrated to determine inclusion or exclusion. Twenty-two abstracts met our inclusion criteria (as defined in “eligibility criteria” above).

**Fig. 1:**
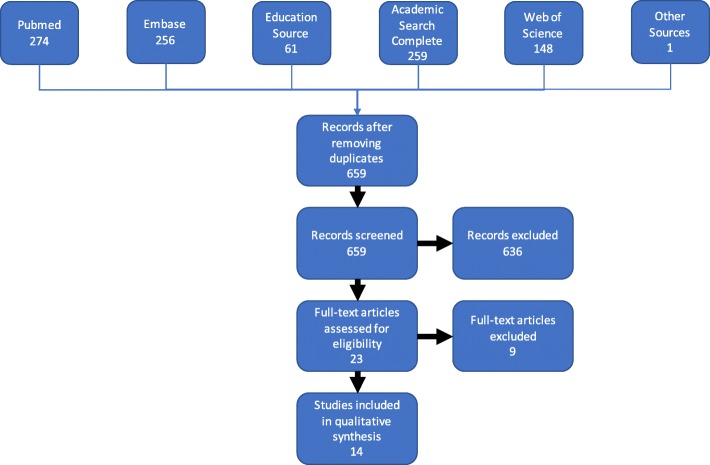
A graphical representation of the inputs and output of our systematic literature search. The number of abstracts and titles identified by systematic search through the five databases the papers identified through personal reading or citations. The flow chart then identifies the records remaining after duplicates were removed, that resulted in 659 titles and abstracts being screened, of which 23 went full-text screening. The final 14 papers that passed the full-text screening were included in our qualitative synthesis

After title and abstract screening, a second round of screening using the same inclusion and exclusion criteria was applied to the full text of each paper with disagreements resolved in the same fashion. The round of full-text screening eliminated nine of the 23 papers. Six were eliminated for not having a published full-text. Three were eliminated for either not including ethical content or not using a research methodology to report the experiences of medical learners (Fig. 1). One additional paper met the inclusion criteria, but was not included in the original search due being published after the search was conducted. The final 14 papers that were included are listed in Table 2.

**Table 2:** Final Papers Included in Systematic Review

Title	Year	Program profession (specialty)	Interviewees	Total Interviewees (n)	Trainees Interviewed about trip experiences (n)	Methods	Method Details
Ethical dilemmas during international clinical rotations in global health settings: Findings from a training and debriefing program [37]	2018	Medicine	Yale medical students participating in international clinical electives	82	43	Mixed-methods	Surveys, combined with free-response, grounded-theory
What are the Ethical Issues Facing Global-Health Trainees Working Overseas? A Multi-Professional Qualitative Study [31]	2016	Medicine, Nursing, Pharmacy	Faculty members at UCSF Schools of Medicine, Nursing, or Pharmacy with experience in global health	18	0	Qualitative	Focus groups with opening questions and unstructured discussion; grounded theory analysis
Medical Electives in Sub-Saharan Africa: A Host Perspective [30]	2015	Medicine	Elective hosts (clinical and administrative staff) in three African countries	14	0	Qualitative	Semi-structured interviews; framework analysis
A Pilot Curriculum in International Surgery for Medical Students [39]	2015	Medicine (Surgery)	Medical students participating in a surgical elective in Northern India	6	6	Qualitative	Reflective essays; grounded theory analysis
Assessment of Ethics and Values During an Interprofessional, International Service Learning Experience [34]	2015	Medicine, nursing, public health, pharmacy, dentistry	Faculty and students enrolled in medical, nursing, public health, pharmacy, and dental school who had participated in service trip to Bolivia	22	3–4^a^	Mixed-Methods	Post-trip survey & journaling prompts. Thematic content analysis to analyze free response text
Global Health Opportunities in Obstetrics and Gynecology Training: Examining Engagement Through an Ethical Lens [35]	2015	Medicine (OB/GYN)	Faculty and staff of OB/GYN residency programs that offer global health experiences	19	0	Qualitative	Semi-structured interviews; unspecified qualitative analysis
Toward Reciprocity: Host Supervisor Perspectives on International Medical Electives [36]	2014	Medicine	Supervisors from 22 countries who had hosted international elective students from Canadian medical schools	39	0	Qualitative	Questionnaires; conventional content analysis
The Ethics and Safety of Medical Student Global Health Electives [38]	2014	Medicine	Canadian medical trainees who had gone on short-term, global health trips	23	23	Qualitative	Semi-structured interviews; transcendental phenomenological analysis
Designing an Ethics Curriculum to Support Global Health Experiences In Surgery [33]	2014	Medicine (Surgery)	Medical students at Emory returning from a trip to Haiti	17	17	Mixed-Methods	35-item questionnaire including free-response survey for ethical concerns
International Surgical Clerkship Rotation: Perceptions and Academic Performance [32]	2013	Medicine (Surgery)	MS3’s and surgeons from Emory who participated in a 1-week elective in Haiti	31	28	Mixed-Methods	27-item questionnaire; followed by free-response survey for specific ethical or safety incidents
Understanding the Effects of Short-Term International Service–Learning Trips on Medical Students [1]	2012	Medicine	First year medical students from U. of Michigan who had participated in an international service project	13	13	Qualitative	Semi-structured interviews, grounded theory analysis
Ethical Issues Encountered by Medical Students During International Health Electives [22]	2011	Medicine	Medical students after returning from a short-term, global health trip	12	12	Qualitative	Semi-structured interviews; inductive data analysis using constant comparison
Canadian Residents Teaching and Learning Psychiatry in Ethiopia: A Grounded Theory Analysis Focusing on their Experiences [40]	2010	Medicine (Psychiatry)	University of Toronto Psychiatry Residents who had gone on a trip to a program in Addis Abbaba	11	11	Qualitative	Semi-structured interviews, grounded theory analysis
International Health Electives: Thematic Results of Student and Professional Interviews [23]	2010	Medicine	Convenience sample of students and professionals who had participated on a prior short-term, global health trip	20	10	Qualitative	Semi-structured interviews, modified grounded theory analysis
Totals				245	166–167		

The titles of the papers analyzed in this systematic review are indicated in the first column, sorted by date published. The column ‘Program Profession’ indicated the professions involved in the program(s) studied in the paper, and the specialty was specified in parenthesis, when applicable. The characteristics of the interviewees, as well as the total number and number of trainees interviewed were included in columns four through six. The methods used in the studies are detailed in the final two columns

^a^The exact number was not given

### Data extraction and analysis

Two authors independently analyzed these 14 papers using the qualitative method of thematic synthesis [29]. First, we performed line-by-line coding of the included papers, highlighting text that described an ethical issue within the papers’ findings, as reported in the abstract, results, discussion, and conclusion sections. Rather than imposing our own conceptualization of topics relevant to ethics, we deferred to each paper’s labelling of ethical issues. The goal at this first step was to describe the meaning and content of the ethical issue from codes that emerged from the data itself. There were no pre-existing codes.

To establish coder reliability, a single paper was coded simultaneously by two authors to generate an initial set of content codes and standardized definitions of those codes. The two authors then coded four more papers independently, and met again to clarify code definitions and coding practices. The rest of the papers were then coded independently. New codes could emerge at this stage. After coding was completed, the two authors met to review each instance of each code in each paper to resolve coding discrepancies by consensus. Data were managed using NVivo (version 11, QSR International).

Next, after the coding was completed, individual codes were reorganized, refined, and grouped into catagories based on a combination of the codes’ conceptual proximity to each other and relationships between codes that emerged from the texts. Reorganization and grouping were informed by the researchers’ background in classical principles of Western medical ethics, including notions of benefit and harm, respect for cultures and individuals, and justice-based concerns (such as sustainability). However, other categories were included as well, including moral distress, concepts of medical tourism, and so on, that emerged from the data.

The third and final analytic step was the most synthetic one. Categories of issues were grouped into four major analytical themes based on in-depth and iterative review of the papers, codes and categories in relation to the motivating research question regarding trainees’ ethical experiences. This step was the most interpretive of the three, as we attempted to explain in a more abstract manner how this thematic synthesis emerged from the codes, which in turn were generated by a close analysis of the texts.

## Results

The thematic synthesis generated four themes: (1) trainees’ concerns over perpetuating medical tourism; (2) struggling to identify and balance the benefits and harms of STINTTs; (3) the complicated trainee *mens* (mind); and (4) ethical situations encountered by trainees. The fourth theme was sub-divided into (a) navigating social and cultural dynamics, (b) trainees’ experiences related to the learner role, and (c) ethical situations not qualifying for other catagories. The codebook with sources and example text is shown in Table 3.

**Table 3:** Themes, Sub-themes, and Codes with Sources and Example Text

Code	Sources	Example Text (not included for every code)
Theme 1: Trainees’ Concerns Over Perpetuating Medical Tourism		
Medical tourism • Sub-code: exploitation	[23, 30, 38]	“Two students also acknowledged elements of medical tourism in their own IHEs. Both described having felt like a medical tourist because of their relatively minimal contributions within the host community.” [23]
Negative view of short-term trips	[23, 38]	“...the majority of participants described the IHE in negative terms.” [23]
Awareness of sustainability	[22, 23, 31–35, 40]	“One student noted, “I believe it is unethical to perform a procedure or provide treatment, then leave without ensuring that adequate medical expertise remains to deal with any complications that arise.” [33]
Concerns that short-term trips are neo-colonialist or exploitive Sub-codes • Fear of imposing Western values • Concerns about exploitation • Concerns with vulnerable populations	[1, 22, 23]	“[W]hen you’re really looking out for your own interests and there’s a huge power and economic differential … there’s a potential for exploitation, and … if you’re not really able to know the local interests, there’s a potential for doing harm.” [1] “They were also anxious not to adopt ‘a paternalistic view of “I know better than you because I come from this more developed country”’. One respondent reported that he ‘really did not want to be remembered as one of these people that come in and impose their values and their experiences’.” [22]
Lack of ethical issues	[35, 37]	“A total of 32% of the programs interviewed reported having no ethical questions or situations.” [35]
Theme 2: Struggling to Identify and Balance the Benefits and Harms of STINTTs		
Perceived benefit (or lack thereof) to community	[1, 22, 23, 30, 32, 34, 35, 38, 39]	“...seven students also perceived that they had a positive effect on the communities they served, by providing clinical care for patients…” [1] “For some reason we thought that we could go over there and [help] these people from a medical point of view but we only had classroom learning…So when people ask about my experience I always try to discourage [medical students] from doing observerships because you can’t really contribute.” [38]
Perceived harms to community	[1, 22, 23, 32, 35–38]	“Although some host supervisors denied the occurrence of any harm, others expressed concern that international elective students may negatively impact the local community in terms of resource use and patient care.” [36]
Perceived benefit to trainee	[1, 22, 23, 30–32, 35, 36, 39, 40]	“All respondents spoke of the personal value of participating in an IHE. All respondents identified a range of benefits, including improving clinical skills, expanding perspectives on illness and poverty, developing international relationships, and exploring potential career choices.” [22]
Perceived harm to trainees	[31, 32, 34, 36–38]	“When I was on the wards in [host site] in January, there were a number of [U.S. Medical School] medical students and residents there and they all had on facemasks and they were the only ones on the ward. None of the other staff were wearing masks. And I was standing there with two medical students from somewhere else and thinking to ourselves, what should we do?. .. The wards are filled with HIV/TB patients, who are coughing” (Medicine Physician). [31]
Theme 3: The Complicated Trainee *Mens* (mind)		
Emotional or moral distress • Sub-code: Debriefing—reflection or discussion	[1, 22, 23, 30–32, 34, 36–39]	“Trainees felt that GHEs presented ethical and psychological challenges, often as a consequence of resource disparity. Trainees primarily experienced guilt when unable to provide care to everyone. For some trainees, these challenges led to signs of poor long-term coping, often exacerbated by poor supervision and inadequate numbers of local staff.” [34] (p67)
Lacking self-awareness	[23]	“In our study, self-identification as a medical tourist was poor despite a consensus among participants of the key aspects of medical tourism.” [23]
Motivations of trainees	[1, 22, 23, 33, 39, 40]	“More than 50% of students cited social justice, to learn and teach with colleagues, an opportunity to function more independently, and travel opportunities as reasons for participation.” [33]
Problematic attitudes in trainees	[30]	“...some students do come with an attitude that because they are Western medical students they will know much more than what our clinical officers know. They will find that they are not right and they have to learn to respect the Malawian clinical officers, respect their decision making.” [30]
Mismatched Expectations	[22, 37, 39]	“Many of the challenges experienced by the students in this study reflect a mismatch between the conditions encountered in low-resource settings, student expectations of the IHE and the support and oversight available from both local and home institutions.” [22]
Trainees’ desires to help in the future	[23, 30, 31, 39]	“Western-trained respondents valued the nurturing of a group of students who may later in their careers return and serve in the region as qualified doctors: ...it’s about what they gain in terms of their future career progression rather than what they give us in [the] short term. So it’s the long-term benefit, not the short-term one.” [30]
Theme 4: Ethical Situations Encountered by Trainees		
Sub-theme 1: Navigating social and cultural dynamics		
*Sub-theme 1A: Power dynamics between locals and visitors*		
Power Imbalance	[1, 23, 31, 37]	“Some participants voiced concerns regarding the troubling power dynamics at some destination. In some instances, trainees who had a “Western education” were perceived as possessing superior knowledge regarding clinical issues. Occasionally, even local professionals who were more experienced than the participants themselves would defer to their opinions.” [31]
Insufficient Attention to Local Priorities and Partners • Sub-code: Competition of interests	[1, 23, 33, 35]	“…students also questioned the value and effects of the service they provided and realized the necessity of engaging in a partnership with the community to ensure that all parties’ interests are represented and met.” [1]
Discriminatory treatment for/against a trainee on the basis of their Western origin	[22, 30, 31, 37]	“Several respondents recounted situations in which patients had requested the student perform a procedure even though there was a more competent local health care worker available (R23). R31 described his discomfort at finding that patients seemed to expect him to be able to help them because he was a Westerner: ‘Every time I walked through a hospital… people would beg me to save their lives. …it was like if they think you’re White or you seem to actually know some things...’” [22]
*Sub-theme 1B: Challenges of navigating different culture*		
Navigating different cultures	[22, 31, 33–35, 37, 39, 40]	“One resident described gaining respect for families and their contribution to patient care; at the same time he believed that encouraging Ethiopian peers to adopt a “Western style of respecting patients’ privacy” was important, as patients typically attend hospital visits with many family members. Participants struggled to synthesize their sensitivity and respect for Ethiopian culture with their own deeply held cultural values.” [40]
Communication difficulties	[22, 23, 32–34, 37, 38]	“However, this issue was compounded by language barriers that made it more difficult for respondents to explain their roles to patients and others. Some respondents expressed discomfort when patients did not understand that they were trainees and were not fully qualified. These respondents described feeling like ‘an imposter’ and found the lack of understanding problematic.” [22]
Autonomy, Respect for Persons Sub-codes: • Difficulty with Consent • Respecting Privacy	[22, 31, 33, 34, 37, 39, 40]	“The most common sources of ethical dilemmas participants identified related to difficulties with truth-telling and establishing informed consent. Participants noted that the concept of informed consent in resource-limited settings was very different to what they were familiar with at home.” [31]
Experiencing Different Professional Norms Sub-code: • Practicing Contrary to Evidence-Based Medicine	[1, 22, 32, 37, 39]	“What became quickly evident was the difference in practice compared with U.S. hospitals as Indian physicians are trained under the British system. Unexpectedly, the hospitals were updated with modern technologies, had a significant community focus, and had an extremely high volume patient influx, with minimal implementation of public health and medical education in schools and clinics.” [39]
Being Seen as Other	[1, 37]	“I always thought that I had some vague connection to lower-income urban communities … [but] … then everyone was like, “Hey, look at the white man playing soccer,” to me. I was kind of taken aback and that kind of changed how I saw myself … [and] … saw my role in the community.” [1]
*Sub-theme 1C: Relationships with team members*		
Realizing that Other Trainees Have Different Values	[1]	“Five students were surprised to find during the planning process or during the ISLTs themselves that their classmates did not always share the same idealistic priorities or standards. For example, one student stated that his concern for the health of people in the Third World or developing countries did not appear to be an educational priority for most of his classmates on the trips.” [1]
Perceptions of team members	[34]	Watzak et al., included questions asking survey-respondents about other team members’ behavior, including whether team members were honest or demonstrated respect to patients. [34]
Sub-theme 2: Trainees’ experiences related to the learner role		
Trainee preparedness	[23, 32–35, 37, 38]	“Medical schools have a responsibility to ensure ethical and safe global health experiences. However, our findings suggest that medical students are often poorly prepared for the ethical and safety dilemmas they encounter during these electives.” [38]
Lack of objectives	[23]	“Our study participants consistently described the lack of educational objectives as a negative aspect of IHEs.” [23]
Mistreatment of trainees [by mentors/advisors]	[37, 38]	“Eventually ethically challenging situations occurred where trainees felt ridiculed for their limited skill even when asked to do a procedure clearly beyond their skill level: “Residents would ask: ‘why don’t you do that thoracotomy?’ ‘Well I am a first year med student. I don’t know how [so] I am not going to do it.’…A lot of times the residents or staff would laugh at you for not knowing how to do certain procedures. It was embarrassing.”” [38]
Practicing Out of Scope	[1, 22, 23, 30, 31, 33–35, 37, 38]	“However, four (40%) trainees did describe situations in which they had been asked to perform procedures or skills beyond their comfort level. One trainee, as a Year 1 medical student, had been asked repeatedly to perform lumbar punctures for the first time on her elective in Africa. She expressed feelings of discomfort about gaining experience at the expense of patients who often did not speak the same language as she did. She described the challenge of repeatedly declining these opportunities: ‘I don’t think everyone [in the host community] was aware of my level of clinical skill prior to going there… I didn’t care how many times I would see a lumbar puncture, I wasn’t going to do one for the first time on an African who couldn’t speak [the same language as me].’ (7 T)” [23]
(Not) Practicing Out of Scope	[30]	“I think some of them [elective students] are frustrated because we will not allow them to do things that they are not qualified to do. This is not a bush hospital; this is a hospital that has been there for 105 years... We are never enough, but I would never put a scalpel in the hand of a student, never, it has never happened.” [30]
Lack of Supervision	[1, 22, 30, 32, 35, 37, 38]	“Without enough local physicians, trainees were faced with managing sick patients independently, beyond their level of training. One student described the scenario of either treating patients without adequate knowledge or letting them suffer and die: “A lot of times I was put in situations where there was somebody bleeding in front of me, and I really didn’t know what to do. So I would just do what I could, and hope…” [38]
Sub-theme 3: Ethical situations not qualifying for other sub-themes		
End of life issues	[33]	No examples were provided in the text, this topic was only briefly mentioned as a survey result. [33]
Corruption	[31]	“Another professional issue that many participants discussed was their perception of direct or indirect exposure to corruption.” [31]
Professionalism	[31, 33]	“...students reported enhanced exposure to the professional obligations of surgeons… Professional obligations first and foremost focused on issues of beneficence and non-maleficence surrounding the short-term nature of the work, especially regarding potential postoperative surgical complications and adequate follow-up.” [33]
Research Ethics	[35]	“Two programs described concerns with the ethical conduct of research; for example, one program mentioning that a resident had been accused of taking protected research material without IRB approval from the host country.” [35]
Truth Telling	[22, 31, 33, 37]	“For example, participants reported cases that compelled them to reconsider their duty to inform the patients of the true nature of their affliction and the circumstances of the proposed treatment.” [31]
Resource Limitation	[22, 23, 30–35, 37–40]	“These students also noted a number of logistical errors such as a shortage of a particular glove size or insufficient sharps containers for there to be one next to each patient bed. The final student noted observing a number of patient care risks resulting from an overwhelmed clinical team, which led to scheduled pain medications not being given at the appropriate time or a wound not being redressed per scheduled orders.” [32]

This table includes the themes, sub-themes, codes, and sub-codes generated from our qualitative analysis. The sources column indicates which papers included a given code or sub-code. A sample of text or a summary of the code is provided in the final column

### Theme 1: Trainees’ concerns over perpetuating medical tourism

This theme captured findings that made evaluative statements, not about particular issues per se, but about the entire enterprise of STINTTs. Most of these statements were negatively toned, that is—they noted the negative impact of STINTTs. Some of these critiques included explicit concerns about perpetuating medical tourism [23, 30] and the related concern over the unsustainable nature of some relationships and interventions [22, 23, 31–35]. In one paper, this was quite striking and illustrated by the following quotations:

“The majority of participants described the IHE in negative terms… Two students also acknowledged elements of medical tourism in their own IHEs. Both described having felt like a medical tourist because of their relatively minimal contributions within the host community” [23].

These general evaluative statements appeared important, because they are relevant to how trainees ethically contextualized their trip participation. This contextualization is a component of the trainee’s overall ethical experience and likely influences how they experience other, more discrete ethical issues and situations.

Importantly, however, the concern over the perpetuation of medical tourism was not universal across studies. In fact, in one study that interviewed directors and administrators of residency training programs, 32% of programs “reported having no ethical questions or situations” [35]. This finding, while isolated, revealed two important issues. First, the finding of concern over perpetuating medical tourism, while dominant, may not be universal. Second, this observation could be partly explained by noting that this particular statistic relied upon program-level reporting, not trainees, to assess ethical questions or situations, which underscores the importance of understanding the difference between the trainees’ experiences and program leadership’s perspective of trainees’ experiences.

### Theme 2: Struggling to identify and balance the benefit and Harms of STINTTs

A consequentialist way of thinking about the ethical experiences of students (and the trips in general) is to document various benefits and harms. Throughout the included studies, this was a pervasive issue, but no paper included a comprehensive analysis of both benefits and harms and a clear conception of “who” experienced them. Via our thematic synthesis, we identified potential harms and benefits both to the community (interpreted both as a collective whole, as well as individual patients who receive care from trainees) and to trainees. We characterized this theme as a struggle, because our synthesis suggested that trainees – perhaps due to their status as trainees and their newness to a particular locale – experienced challenges in assessing benefits and harms.

The effects on the community were varied and sometimes nuanced. Some students felt that they did have a positive effect on the communities that they went to serve, in part through non-clinical benefits such as giving respect and attention to patients who otherwise might not have received any [1]. A few studies included perspectives beyond those of the trainees. For example, Watzak et al [34] noted that local communities reported satisfaction and appreciation for the care provided. Another study found that administrators and staff who hosted visiting medical trainees viewed “good mature” medical students as a boon [30]. A chief concern regarding harm to the community was that supporting visiting trainees would consume limited resources, such as faculty attention for teaching or translation [22, 35–37]. Other trainees were concerned that they might impact patient care negatively, through language or clinical skill deficits [22, 23, 38]. On the other hand, many trainees felt that their presence neither benefited nor harmed the community they visited, noting that their limited clinical abilities left them feeling more like spectators than actors capable of creating harm or benefit [1, 22, 23, 34, 38, 39].

As far as the benefits to the trainees themselves, a wide variety of benefits to trainees were described, including gaining clinical skills, [1, 35, 39] language abilities, [1] or a better understanding of healthcare systems [1, 22, 23, 36, 39]. Other students highlighted the role STINTTs played in their professional development and desire to pursue a career in global health [23, 39].

Regarding harms to trainees, some trainees felt that risks to themselves were well-controlled [32]. Others reported physical and emotional threats, including a lack of health safety equipment such as N-95 masks [31, 36–38]. One particularly egregious incident involved a student reporting sexual assault [38].

The following quotation illustrates the difficulties trainees experience in identifying harms and benefits as well as in balancing them across themselves and others:

“…there were a number of [U.S. Medical School] medical students and residents there and they all had on facemasks and they were the only ones on the ward. None of the other staff were wearing masks. And I was standing there with two medical students from somewhere else and thinking to ourselves, what should we do? … The wards are filled with HIV/TB patients, who are coughing” [31].

### Theme 3: The complicated trainee *mens*

A third theme was synthesized from descriptions of trainee’s diverse, and sometimes conflicting internal cognitive and affective components of trainees’ experiences. That is, the trainee *mens* (mind).

Motivation, intent, and attitudes are key parts of the mind, and here trainees expressed diverse perspectives. Some trainees reported experiencing personal maturation on the trip or the strengthening of a desire to return to global health work in the future [39]. In contrast, one of the papers that investigated the perception of hosts reported an observation that some students arrived with problematic attitudes including thinking of the trip as a holiday, occasionally skipping clinical duties, or coming in with an air of clinical superiority to the hosts [30]. Two papers reported that trainees had expectations that did not match with their actual experiences [22, 39].

Mixed motives or problematic attitudes could be compounded by lack of self-awareness about them. For example, although theme 1 above emphasized trainee awareness and concern over perpetuating medical tourism, self-awareness that the trainee had actually participated in just that could be lacking. As one study documented, “…self-identification as a medical tourist was poor despite a consensus among participants of the key aspects of medical tourism” [23].

A significant finding within this theme related to emotional or moral distress that trainees experienced. One manifestation of this distress was a feeling of guilt derived from either impeding patient care or not being able to provide more help to patients [22, 37–39]. Other manifestations included witnessing poor clinical outcomes, often due to a lack of resources, [38] or feeling overwhelmed by the clinical responsibilities [32]. Feelings of moral distress were not limited to medical trainees, but also involved nurses, pharmacists, and physicians when included in the study population [31].

### Theme 4: Ethical situations encountered by trainees

The bulk of the codes in our analysis related to situations—tropes or repeated scenarios—that commonly recurred in the context of STINTTs. We divided this theme into (a) navigating social and cultural dynamics, (b) trainees’ experiences related to the learner role, and (c) ethical problems not qualifying for other catagories.

#### (a) Navigating social and cultural dynamics

This sub-theme could be further divided into codes describing power dynamics and challenges navigating a different culture.

Trainees reported noticing a power imbalance between hosts and visitors, [1, 23, 31, 37] which manifested in multiple ways. Some trainees were concerned about working with vulnerable populations [1] and wanted to avoid exploiting those populations “for one’s own clinical development” [23]. Other trainees reported concerns that their respective STINTTs might not have adequately taken the priorities of their host communities into account [1, 23, 35].

Another interface regarded the treatment of visiting trainees relative to the treatment of trainees at the host site. Some trainees reported feeling that they were given more attention, recognition, or even clinical deference than their level of training warranted because they were from Western countries [22, 31, 37]. In some instances, trainees were asked by local patients to perform procedures instead of local, more-experienced providers simply because they were foreigners [22]. A contrasting perspective was that of host staff at a particular institution who noted that visiting trainees were less of a priority than local trainees [30].

The second group of codes described trainees’ difficulties with navigating different cultures. Two of the most common points of cultural clash arose with different expectations and practices over what it meant to respect patient privacy and confidentiality [22, 31, 33, 37, 39, 40] and to obtain informed consent [1, 22, 31, 33, 34, 37].

#### (b) Trainees experiences related to the learner role

The most frequently reported experiences encountered by trainees were (1) lack of adequate supervision and (2) requests for trainees to practice outside of their scope of their clinical proficiency [1, 22, 23, 31–33, 35, 37, 38]. For example, Petrosoniak et al. [23] reported first year medical students being asked to perform lumbar punctures or to close after surgical procedures—in both instances the trainee was expected to perform these tasks without supervision. Zaidi et al. [35] describe a situation in which a resident was abruptly left in charge of overseeing patient care for several hours at a hospital. In their own, raw words, trainees recount such challenges:

“A lot of times I was put in situations where there was somebody bleeding in front of me, and I really didn’t know what to do. So I would just do what I could, and hope [I was] doing the right thing. A lot of times there was just nobody to help you and so you are all by yourself.” [38].

“You feel bad for the patient…Here we are [myself and a local trainee] learning how to do an LP on [a patient] and this is both of our first time. There is no staff to teach you…You get into a very sticky situation. It is either me doing it for the first time or that resident. You felt terrible as a human being [thinking] ‘Am I really going to help this person? No. Most likely they will die from an infection because I am doing something wrong but there is nobody to teach me.’ ” [38]

Referring to other team members, one trainee stated, “I think some people were definitely more [under] the impression that … it was okay to … [practice certain skills] even though they had no idea what they were doing…” [1]

Negotiating discussions about trainees’ level of skill was difficult, with trainees often having to address misunderstandings from hosts about their level of training [22, 23, 38]. Sometimes trainees would experience negative consequences—including feeling like an imposter, being repeatedly asked to practice outside of their scope despite having previously explained their refusal, or even being humiliated by host staff—even if they felt they were doing the right thing by refusing to participate in the procedure [22, 23, 38]. For example:

“Residents would ask: ‘why don’t you do that thoracotomy?’ ‘Well I am a first year med student. I don’t know how [so] I am not going to do it.’…A lot of times the residents or staff would laugh at you for not knowing how to do certain procedures. It was embarrassing” [38].

Notably, not all papers shared the same perspective on this topic. In a paper describing host perspectives, [30] one host staff denied that trainees were allowed to perform procedures beyond their scope of practice, while others acknowledged that supervision was indeed limited. Watzak et al., [34] while not reporting directly from trainee experiences, noted that trip organizers carefully assigned tasks to trainees appropriate to their ability. Leeds et al. [32] reported that 78% of their participating trainees thought that supervision on their trip was adequate.

Finally, many papers commented on the preparedness of trainees to participate on these trips with some suggesting that trainees were not ethically equipped to handle the situations they faced on a STINTT due to lack of pre-departure training [23, 32, 38].

#### (c) Ethical problems not qualifying for other catagories

There were several ‘familiar’ ethical problems that overlapped with those encountered in everyday Western clinical settings including end of life issues, [33] navigating professional obligations, [33] truth telling, [31, 33, 37] and ethical conduct of research [35].

Other ethical problems that did not directly fit into other catagories, but that were nonetheless important, included encountering corruption [31] and providing clinical care in the context of limited resources. Resource limitations were reported in many of the studies and sometimes negatively impacted patient care [22, 23, 31–35, 37–40]. Some papers mentioned the benefit to trainees of learning how to practice medicine with heightened awareness of resource use [35, 40].

## Discussion

Our systematic qualitative synthesis produced the following principal findings. First, our findings demonstrate that the peer-reviewed literature documenting what medical trainees experience during STINTTs is relatively nascent. All 14 studies were published after 2010. Most studies were qualitative, with only a few mixed-method studies, and the entirety of the literature represents the experiences of less than 170 medical trainees. In addition, many studies investigated STINTTs from a single institution to a single host site abroad [32–34, 39, 40]. In several studies, trainees’ experiences were reported indirectly, for instance, by educators, administrators, trip leaders, or trip hosts [30, 31, 35, 36].

Second, our review found that medical trainees report experiencing a variety of ethical challenges on STINTTs. Many of these have been recognized by prior case studies, editorials, and best practice guidelines. These included concerns about intervention sustainability, benefits and harms of STINTTs (both to trainee and host), trainee moral distress, trainee motivations, trainee preparedness and supervision (or lack thereof), the challenges of navigating different cultures, and facing resource limitations [5, 10, 16, 17, 37, 41, 42].

At the same time, we found several issues not well reflected in this prior literature. These included overt mistreatment of medical trainees and unique ethical issues regarding perceptions of, and relationships with, other STINTT team members. Ethical challenges occurring during STINTTs may not always be experienced by individuals in isolation; instead they may be experienced within teams, and these teams can include trainees of various professional backgrounds (e.g., medical students, nurses, physician assistants, pharmacists, and others). Some educators have already touted the importance of interprofessional ethics education [43–45]. Interprofessional curricula might need to be developed for the STINTT setting as well.

A comprehensive comparison of our findings to those of a recent systematic review of the ethics topics addressed by ethics education for STINTTs would be beyond the scope of this study. Nevertheless, we can make several observations about the relative fit between existing ethics education (See Table 4 in Rahim et al) [25] and our findings. In general, our findings suggest that existing ethics education covers many, but not all, of the issues trainees report experiencing. For instance, ethical challenges related to demonstrating respect for other cultural norms, obtaining appropriate informed consent, dealing with limited resources, and practicing beyond one’s level of training are addressed by existing curricula [25]. In contrast, ethical challenges related to the emotional and moral distress trainees face, overall sustainability of STINTTs, and navigating medical, cultural, and social hierarchies might not be well reflected in existing ethics curricula. Educators should consider the inclusion of these topics in their pre-departure curricula.

Our findings identify a few areas requiring emphasis from medical educators. Aside from the need to develop interprofessional curricula, educators might need to attend explicitly to trainees’ distress. Some of the most vivid experiences trainees reported involved emotional or moral distress (theme 3). Educators should aim to prepare trainees for distressing situations, prevent distressing experiences when possible (e.g. by adhering to existing best practice guidelines, [5] and alleviate distress when it arises. Preparation involves knowing the types of distressing experiences that trainees face and fore-warning them pre-departure as well as giving them strategies for addressing situations should they arise. Instituting mandatory debriefing for all trainees on STINTTs might help alleviate distress through group reflection [14, 22]. Debriefing can also help educators identify trainees who need further support and connect them to further resources.

Some of the situations encountered by trainees were extremely complex and involved difficulties with communication, expectations, language barriers, and cultural and hierarchical norms (theme 4a and 4b). Trainees could be prepared for common experiences, such as being asked to do something out of one’s scope of training, by using existing predeparture training resources [46] and strategies, [25] including simulations (a pedagogy recently introduced to pre-departure training) [16]. Educators should counsel trainees on how to handle these situations in the specific context where trainees will be. Some experiences cannot be foreseen, and access to an onsite faculty mentor can be a critical strategy to address these situations [14]. Such faculty could serve as a supervisor, mentor, or liaison depending on the situation. Trip planners should provide all trainees with a way to contact a mentor. Long-term partnerships between hosting and sending sites are favorable because they allow for stable training experiences and matured expectations.

Preparing trainees to navigate ethical issues in STINNTs cannot be accomplished entirely by pre-departure training; program leadership should explore other avenues to minimize trainees’ vulnerability. A comprehensive approach to management also requires attention to the appropriate structuring of organizations and programs that serve as the vehicle for trainees’ experiences abroad [47]. For example, preparation may help trainees respond to requests to performs skills outside one’s scope of training, but organizations and programs should make efforts to ensure expectations are clear by engaging in open dialogue with local communities about what skills trainees do and do not have. Similarly, organizations and programs are primarily responsible for protecting trainees from risk of harm by providing appropriate precautions (e.g., protective equipment).

Our study has several limitations. First, in the absence of controlled vocabulary or unified definitions of STINTTs, the application of our search terms may have excluded other relevant studies. Second, in line with our principal findings (which highlight what may be a young and rapidly growing field), our search end date of December 2016 will soon be outdated. Third, the qualitative nature of our study and many of the studies we reviewed means that our findings, while systematically structured, are qualitatively subjective. This is the nature of qualitative research; in our study, for instance, we chose to characterize some text as being about ethics, even if the primary article did not explicitly frame that text as an ethical issue (see Methods: Data Extraction and Analysis for more details). Fourth, our review was geographically limited to LCME, CACMS, ACGME, and RCPSC-accredited programs within the U.S. and Canada. While these countries represent the two largest nations of origin for medical short-term international trips, [2] findings from other countries’ training programs were not included. Lastly, we excluded anecdotal reports and case studies. This focused the review on the unique perspective of trainees’ experiences as directly reported in quantitative and qualitative research studies, but could have missed other issues and rich insights from these alternative data sources [48].

Nevertheless, we believe our review has important implications for future research into the ethical challenges medical trainees face when engaging in STINTTs and for curricular design. First, future qualitative studies of trainees’ experiences abroad should focus not just on what challenges they experience, but how they manage them. For example, further qualitative documentation that trainees experience pressure to exceed their level of training abroad may no longer be necessary; instead, attention should be given to how trainees navigate these situations (e.g., what works and what does not). Second, the time may be right to move toward quantitative research methods, such as surveys, that document the epidemiology of these challenges, i.e., how often they occur and under what circumstances. Third, by systematically documenting what trainees report experiencing, our findings should inform development of future ethics education curricula before, during, and after STINTTs.

## Conclusion

Medical trainees report experiencing a wide range of ethical challenges during short-term international trips in which they engage in clinical or research activities. The literature covering US and Canadian STINTTs is relatively young and largely qualitative. However, emerging consensus about the nature of ethical challenges experienced and existing best practice and ethics guidelines suggest that this area of inquiry is at an inflection point. Future research should focus on quantitative studies that examine the relative frequency of challenges experienced and qualitative studies that probe more deeply into how best to manage them.

## Appendix

**Table 4:** Enhancing transparency in reporting the synthesis of qualitative research (ENTREQ) checklist

No	Item	Guide and description	Comment
1	Aim	State the research question the synthesis addresses.	See Abstract and Background
2	Synthesis methodology	Identify the synthesis methodology or theoretical framework which underpins the synthesis, and describe the rationale for choice of methodology *(*e.g. *meta-ethnography, thematic synthesis, critical interpretive synthesis, grounded theory synthesis, realist synthesis, meta-aggregation, meta-study, framework synthesis).*	Thematic synthesis
3	Approach to searching	Indicate whether the search was pre-planned (*comprehensive search strategies to seek all available studies)* or iterative (*to seek all available concepts until they theoretical saturation is achieved)*.	Pre-planned, as identified in Table 1 and Methods
4	Inclusion criteria	Specify the inclusion/exclusion criteria *(*e.g. *in terms of population, language, year limits, type of publication, study type).*	Detailed under the heading entitled, “Eligibility criteria” in Methods
5	Data sources	Describe the information sources used (e.g. *electronic databases (MEDLINE, EMBASE, CINAHL, psycINFO, Econlit), grey literature databases (digital thesis, policy reports), relevant organisational websites, experts, information specialists, generic web searches (Google Scholar) hand searching, reference lists)* and when the searches conducted; provide the rationale for using the data sources.	Pubmed, Embase, Education Source, Academic Search Complete, and Web of Science (Core Collection), plus reference list searches and the authors’ own knowledge of manuscripts. See Methods
6	Electronic Search strategy	Describe the literature search *(*e.g. *provide electronic search strategies with population terms, clinical or health topic terms, experiential or social phenomena related terms, filters for qualitative research, and search limits)*.	See Table 1
7	Study screening methods	Describe the process of study screening and sifting *(*e.g. *title, abstract and full text review, number of independent reviewers who screened studies).*	See Methods and Figure
8	Study characteristics	Present the characteristics of the included studies *(*e.g. *year of publication, country, population, number of participants, data collection, methodology, analysis, research questions).*	See Table 2
9	Study selection results	Identify the number of studies screened and provide reasons for study exclusion *(e, g, for comprehensive searching, provide numbers of studies screened and reasons for exclusion indicated in a figure/flowchart; for iterative searching describe reasons for study exclusion and inclusion based on modifications t the research question and/or contribution to theory development).*	See Methods and Figure
10	Rationale for appraisal	Describe the rationale and approach used to appraise the included studies or selected findings *(*e.g. *assessment of conduct (validity and robustness), assessment of reporting (transparency), assessment of content and utility of the findings).*	See Methods and Table 2 for our appraisal of the studies’ methods and scope. Content was appraised as a part of the screening process, as detailed in the heading titled, “study selection.”
11	Appraisal items	State the tools, frameworks and criteria used to appraise the studies or selected findings *(*e.g. *Existing tools: CASP, QARI, COREQ, Mays and Pope* [25]*; reviewer developed tools; describe the domains assessed: research team, study design, data analysis and interpretations, reporting).*	Domains assessed included the study design (qualitative vs. mixed-methods), study size (n), interviewees, and content. See Methods and Table 2 for further details.
12	Appraisal process	Indicate whether the appraisal was conducted independently by more than one reviewer and if consensus was required.	Appraisal of the methodology, interviewees, and study size was conducted by a single reviewer, and appraisal of content was conducted by two reviewers independently as part of the screening process. See Methods for further details.
13	Appraisal results	Present results of the quality assessment and indicate which articles, if any, were weighted/excluded based on the assessment and give the rationale.	No studies were excluded based on results of the appraisal, and the appraisal of ‘n’ and the qualitative nature of most of the studies are commented on in Methods and Discussion.
14	Data extraction	Indicate which sections of the primary studies were analysed and how were the data extracted from the primary studies? *(*e.g. *all text under the headings “results /conclusions” were extracted electronically and entered into a computer software).*	The sections of the studies analyzed were *abstracts*, *results*, *discussion*, and *conclusion*. See Methods heading, “Data Extraction and Analysis.”
15	Software	State the computer software used, if any.	NVivo
16	Number of reviewers	Identify who was involved in coding and analysis.	This is detailed in Methods
17	Coding	Describe the process for coding of data *(*e.g. *line by line coding to search for concepts).*	See Methods: coding was line by line.
18	Study comparison	Describe how were comparisons made within and across studies *(*e.g. *subsequent studies were coded into pre-existing concepts, and new concepts were created when deemed necessary).*	Line-by-line codes were coded into pre-existing concepts and new concepts were created if deemed necessary.
19	Derivation of themes	Explain whether the process of deriving the themes or constructs was inductive or deductive.	We derived the codes and major themes inductively, with no a priori codes.
20	Quotations	Provide quotations from the primary studies to illustrate themes/constructs, and identify whether the quotations were participant quotations of the author’s interpretation.	Provided in Results
21	Synthesis output	Present rich, compelling and useful results that go beyond a summary of the primary studies (e.g. *new interpretation, models of evidence, conceptual models, analytical framework, development of a new theory or construct).*	See Discussion.

## Abbreviations

ACGME: Accreditation Council for Graduate Medical Education
CACMS: Committee on Accreditation of Canadian Medical Schools
ENTREQ: Enhancing transparency in reporting the synthesis of qualitative research
LCME: Liaison Committee on Medical Education
PRISMA: Preferred Reporting Items for Systematic Reviews and Meta-Analyses
RCPSC: Royal College of Physicians and Surgeons of Canada
STINTTs: short-term international trips for trainees (STINTTs)
